# Correlative Detection of Isolated Single and Multi-Cellular Calcifications in the Internal Elastic Lamina of Human Coronary Artery Samples

**DOI:** 10.1038/s41598-018-29379-6

**Published:** 2018-07-20

**Authors:** Han Wen, Alejandro Morales Martinez, Houxun Miao, Thomas C. Larsen, Catherine P. Nguyen, Eric E. Bennett, Kellan P. Moorse, Zu-Xi Yu, Alan T. Remaley, Manfred Boehm, Ahmed M. Gharib

**Affiliations:** 1 National Heart, Lung and Blood Institute, National Institutes of Health, Bethesda, MD 20892 USA; 2 Department of Bioengineering, University of California, Berkeley, CA 94720 USA; 3 National Institute of Diabetic and Digestive and Kidney Diseases, National Institutes of Health, Bethesda, MD 20892 USA

## Abstract

Histopathology protocols often require sectioning and processing of numerous microscopy slides to survey a sample. Trade-offs between workload and sampling density means that small features can be missed. Aiming to reduce the workload of routine histology protocols and the concern over missed pathology in skipped sections, we developed a prototype x-ray tomographic scanner dedicated to rapid scouting and identification of regions of interest in pathology specimens, thereby allowing targeted histopathology analysis to replace blanket searches. In coronary artery samples of a deceased HIV patient, the scanner, called Tomopath, obtained depth-resolved cross-sectional images at 15 µm resolution in a 15-minute scan, which guided the subsequent histological sectioning and microscopy. When compared to a commercial tabletop micro-CT scanner, the prototype provided several-fold contrast-to-noise ratio in 1/11^th^ the scan time. Correlated tomographic and histological images revealed two types of micro calcifications: scattered loose calcifications typically found in atherosclerotic lesions; isolated focal calcifications in one or several cells in the internal elastic lamina and occasionally in the tunica media, which we speculate were the initiation of medial calcification linked to kidney disease, but rarely detected at this early stage due to their similarity to particle contaminants introduced during histological processing, if not for the evidence from the tomography scan prior to sectioning. Thus, in addition to its utility as a scouting tool, in this study it provided complementary information to histological microscopy. Overall, the prototype scanner represents a step toward a dedicated scouting and complementary imaging tool for routine use in pathology labs.

## Introduction

In histopathology protocols, the process of serial sectioning, slide staining and microscopy are often laborious and involve trade-offs between workload and the sampling density of the sections^1–5^. Therefore, tabletop micro-CT systems have been used to obtain 3D scouting images to guide the sectioning and microscopy to regions of interest^6^. Micro-CT has been a valuable imaging tool in pathology studies, as exemplified by a few examples of a vast literature^6–28^. However, the scan time is on the order of several hours with commercial micro-CT systems unless the bright x-ray beams of synchrotron facilities are used, which is illustrated by a few examples of a multitude of high-quality studies from synchrotron beamlines^29–35^. Therefore, the long scans required by benchtop systems make routine use difficult. As a solution to this problem we investigated a prototype x-ray tomographic scanner dedicated to histopathology (Tomopath), which obtained depth-resolved cross-sectional images of paraffin-embedded samples in 15-minute scans.

This study of the coronary artery samples of a deceased HIV patient was part of an ongoing clinical study of patients with HIV who develop early coronary artery disease in order to understand the nature of atherosclerosis in this patient population^36^. Due to a few design optimizations tailored to histopathology samples, Tomopath provided sufficient resolution in paraffin-embedded coronary segments to detect micro calcifications as small as a single cell, and sufficient tissue contrast to show the vessel wall layers in most cases, thereby allowing matching with the subsequent histological images and placement of the micro calcifications in the layered structure of the coronary wall. After the Tomopath scan, the paraffin block was sectioned to targeted levels, stained for soft tissue structure and calcium deposits, and scanned in a digital slide scanner. Correlated findings between the tomographic and histological images confirmed two forms of micro calcification deposits that were present in the coronary artery of the patient.

## Results

### Tomographic images of the coronary artery samples

**Figure 1:**
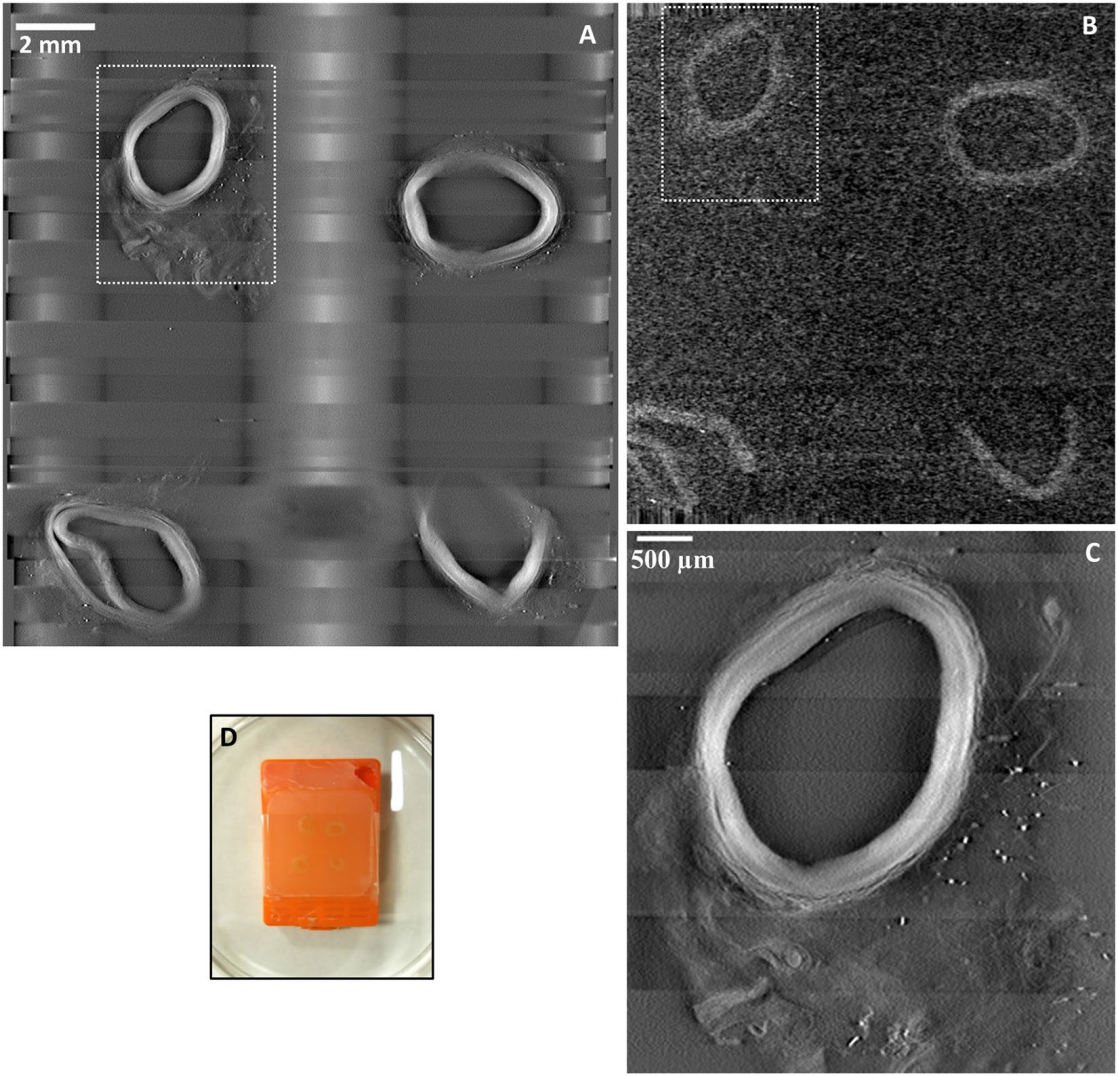
Comparing a prototype tomographic scanner dedicated to histopathology (Tomopath) with a commercial tabletop micro-CT scanner. (**A**) Cross-sectional image at 1.3 mm level below the surface of a paraffin block from the Tomopath scanner. Left-anterior-descending (LAD) coronary artery segments of a deceased HIV patient are embedded in the block. The vessel walls appear bright against a dark background from the x-ray absorption contrast between tissue and paraffin medium. The grid-like shadows are cast by the plastic grid of the embedding cassette. (**B**) Cross-sectional image at the same level from the commercial tabletop micro-CT scanner. (**C**) Zoomed-in view of the top left LAD segment outlined in A from the Tomopath scanner. The layers of the vessel wall are visible. The scan times of the micro-CT and the Tomopath scanners were 2.75 hours and 15 minutes, respectively. (**D**) Picture of the intact paraffin block prior to histological sectioning.

Movies of serial cross-sectional images through the thickness of a paraffin block are provided as Supplementary Information Movie S1 for the Tomopath scanner and Movie S2 for the commercial tabletop micro-CT. Image quality from both scanners was assessed visually and quantitatively by measuring the contrast-to-noise ratio. Figure 1 shows images of matching levels from the two scanners. In terms of the measured contrast-noise-ratio between the vessel wall and the surrounding paraffin medium, the Tomopath scanner was 3.9 times the tabletop micro-CT (7.5 ± 1.9 vs 1.9 ± 0.3). Figure 1C illustrates that the image resolution is visibly higher with the Tomopath scanner and allowed differentiation of the layers within the vessel wall.

### Correlative detection of two forms of calcification

**Figure 2:**
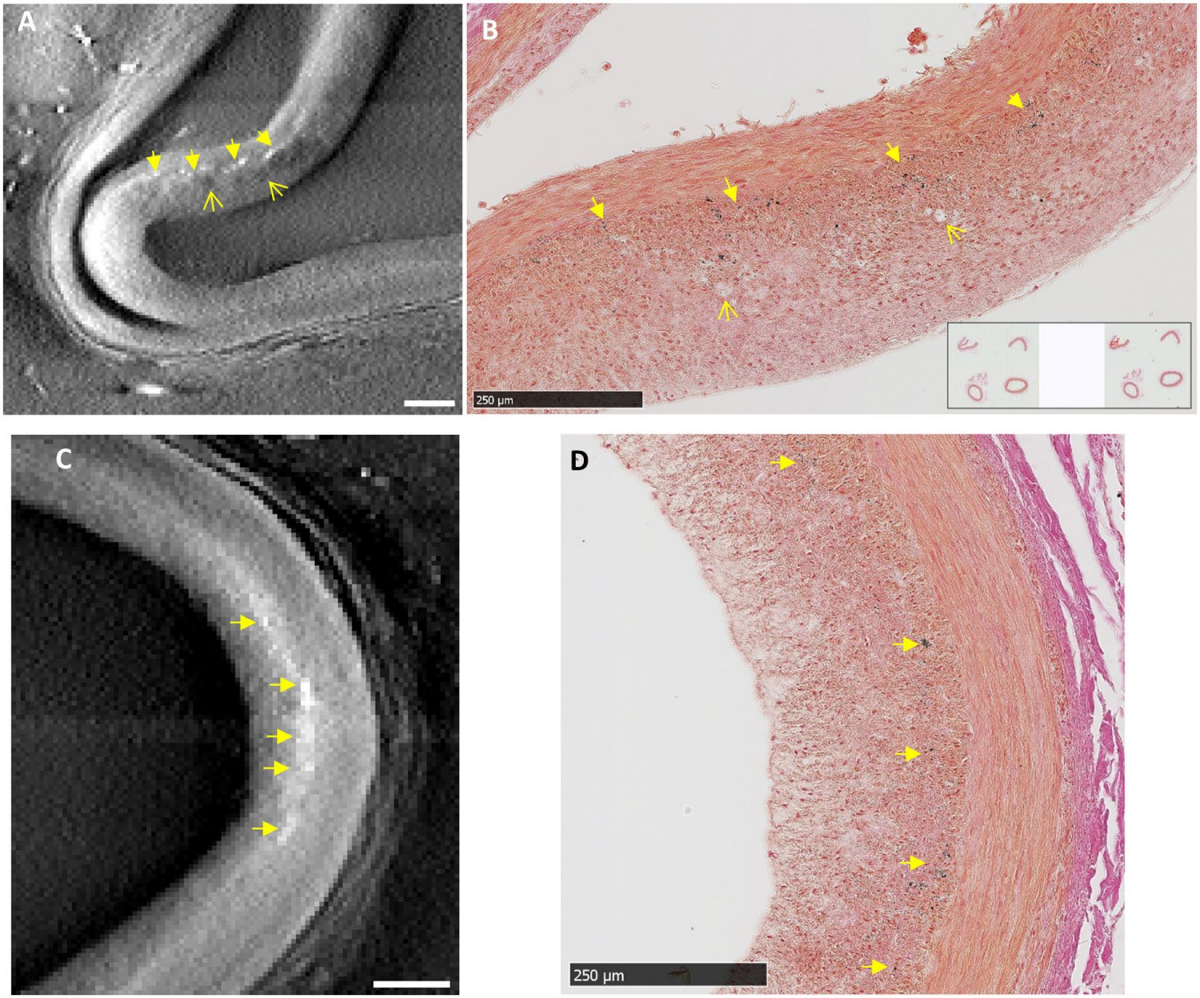
Correlated findings of loose clusters of micro calcifications in the intima in location-matched pairs of tomographic and histological images. In x-ray tomographic images calcifications appear as brighter dots and patches due to their higher mass density; adipose tissue such as foam cells appear as dark patches due to their lower density. The histological slides were stained with von Kossa stain for calcifications which appear as dark brown or black color. (**A**) and (**B**) Scattered clusters of micro calcifications (solid arrows) of an atherosclerotic lesion are seen in matching locations of the paired images, together with foam cells in the neo intima (open arrows). (**C**) and (**D**) A second lesion shows scattered intimal micro calcifications (solid arrows) in matching locations between the x-ray and histological images. Scalebars are 250 µm.

Careful evaluation of the tomographic and histology images at the same sectioning levels revealed two forms of micro calcifications. Since calcifications are denser and more x-ray absorbent than the surrounding tissue, they appear as brighter dots or patches in the tomographic images. The histology slides were stained with either H&E or von Kossa stains. With von Kossa stain, calcifications appear as dark-colored (black) silver precipitates, while tissue structures are also stained to a lighter degree. The first form of calcification was loose clusters of micro deposits in the intima, which typically occur in atherosclerotic lesions. Figure 2 shows the correlated images of this type of calcification from tomography and histology. In the tomographic image of Fig. 2A, dark patches are also seen in the intima around the calcifications, indicating tissue of lower density. These were identified as the fatty foam cells in the neo intima at the lesion, which are visible in the histological image of Fig. 2B. Figure 2C,D are another example of scattered clusters of calcifications in the intima, which are seen in matching locations in the x-ray and histological images.

A second form of calcification observed in the samples were isolated, focal micro calcifications. These were predominantly in the internal elastic lamina (IEL) and occasionally in the tunica media. The individual micro calcification particles were larger than the particles in the first form of calcification, ranging from one to several cells in the histological images (5 to 25 µm), and one to several pixels in the x-ray images. Figure 3 is a detailed example of a focal calcification in the IEL, which was visible at the same location in the tomographic and histological images. Its IEL origin was confirmed by the histological image at the highest magnification (Fig. 3E). Figure 4 summarizes 8 examples of correlated detection of focal calcifications in the IEL by tomography and histology. Histology showed that their sizes ranged from one to several cells in the IEL.

**Figure 3:**
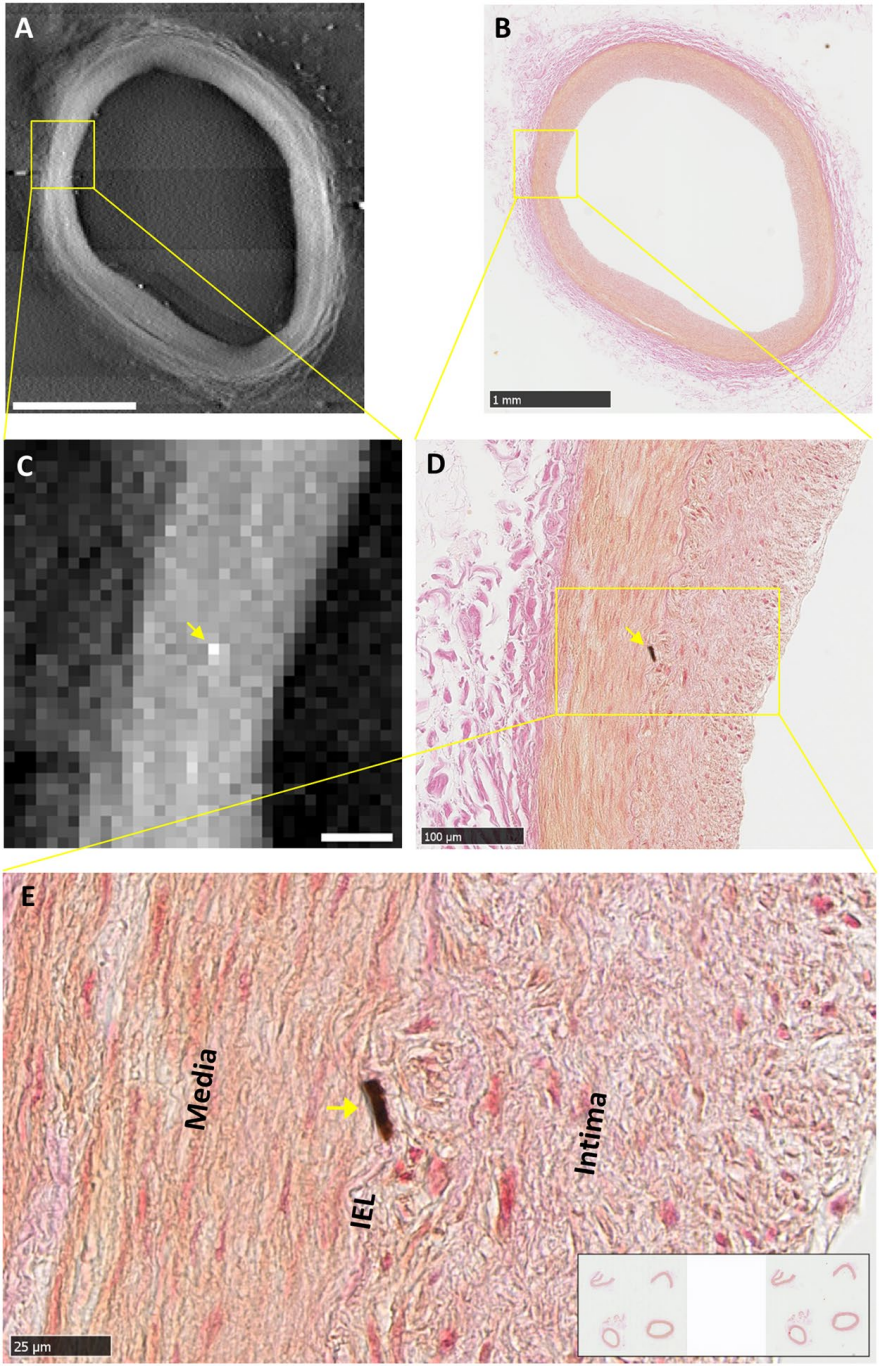
An example of correlated finding of an isolated, focal micro calcification in the internal elastic lamina (IEL). (**A**) Tomographic cross-sectional image of the intact coronary segment shows a bright dot in mid-wall as outlined in square. Scalebar is 1 mm. (**B**) Histological image of the section at the same level in low magnification shows a faint brown stain at the same location outlined in square. Scalebar is 1 mm. (**C**) Magnified view of the outlined area in panel A shows a bright (high density) dot. Scalebar is 100 µm. (**D**) Magnified view of the corresponding area in the histological image shows a focal calcification at the same location, which appears dark brown by the von Kossa stain. Scalebar is 100 µm. (**E**) Further magnification of the histological image shows the location of the micro-calcification in the IEL. Scalebar is 25 µm.

**Figure 4:**
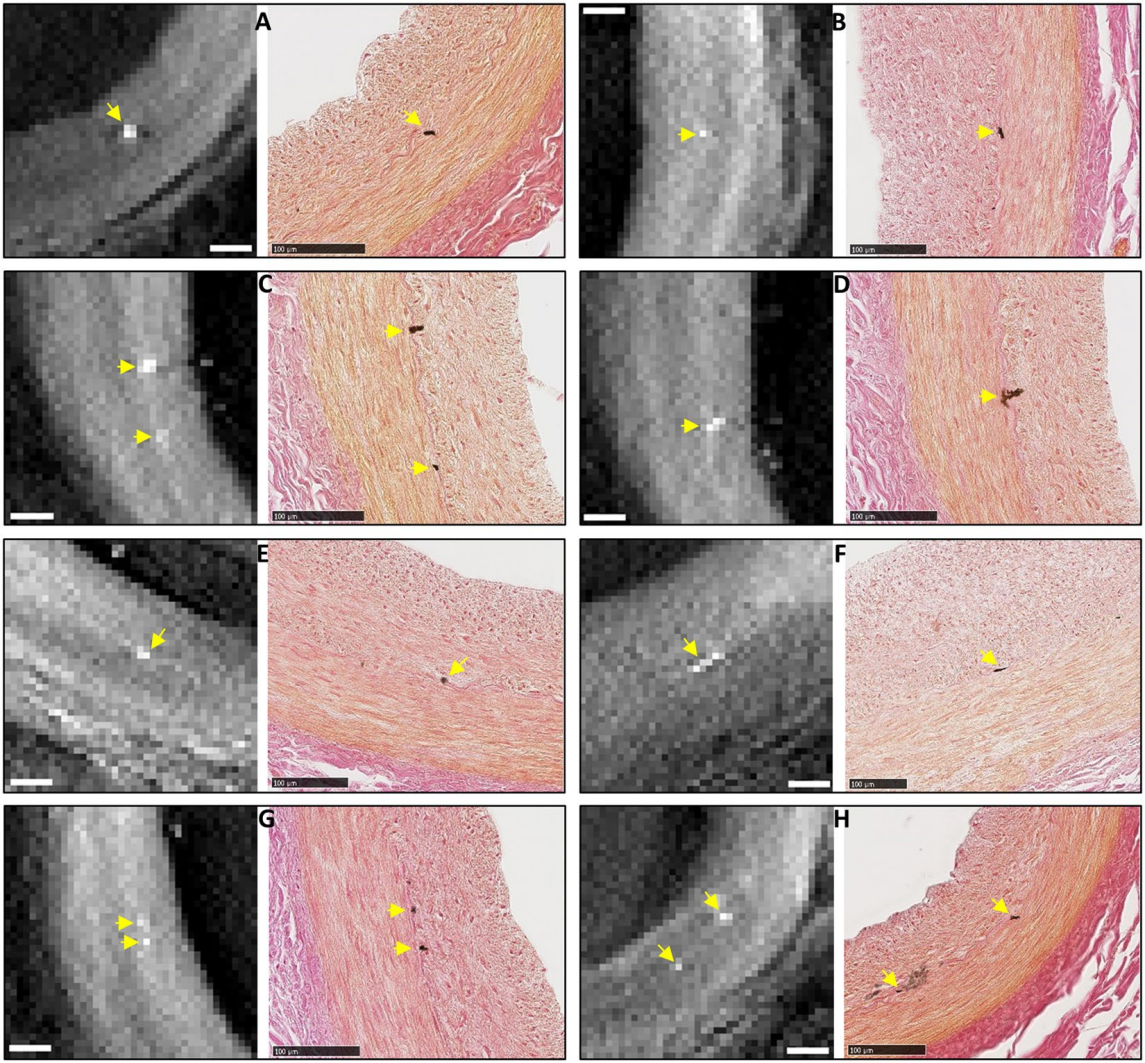
A collection of 8 examples of correlated findings of focal micro calcification in the IEL of the coronary artery samples of the patient. Each panel contains a tomographic cross-sectional image on the left and the matching histological micrograph on the right. Micro calcifications are marked by yellow arrows. The histological images confirm the locations of the calcifications to be in the IEL. All scalebars are 100 µm.

**Figure 5:**
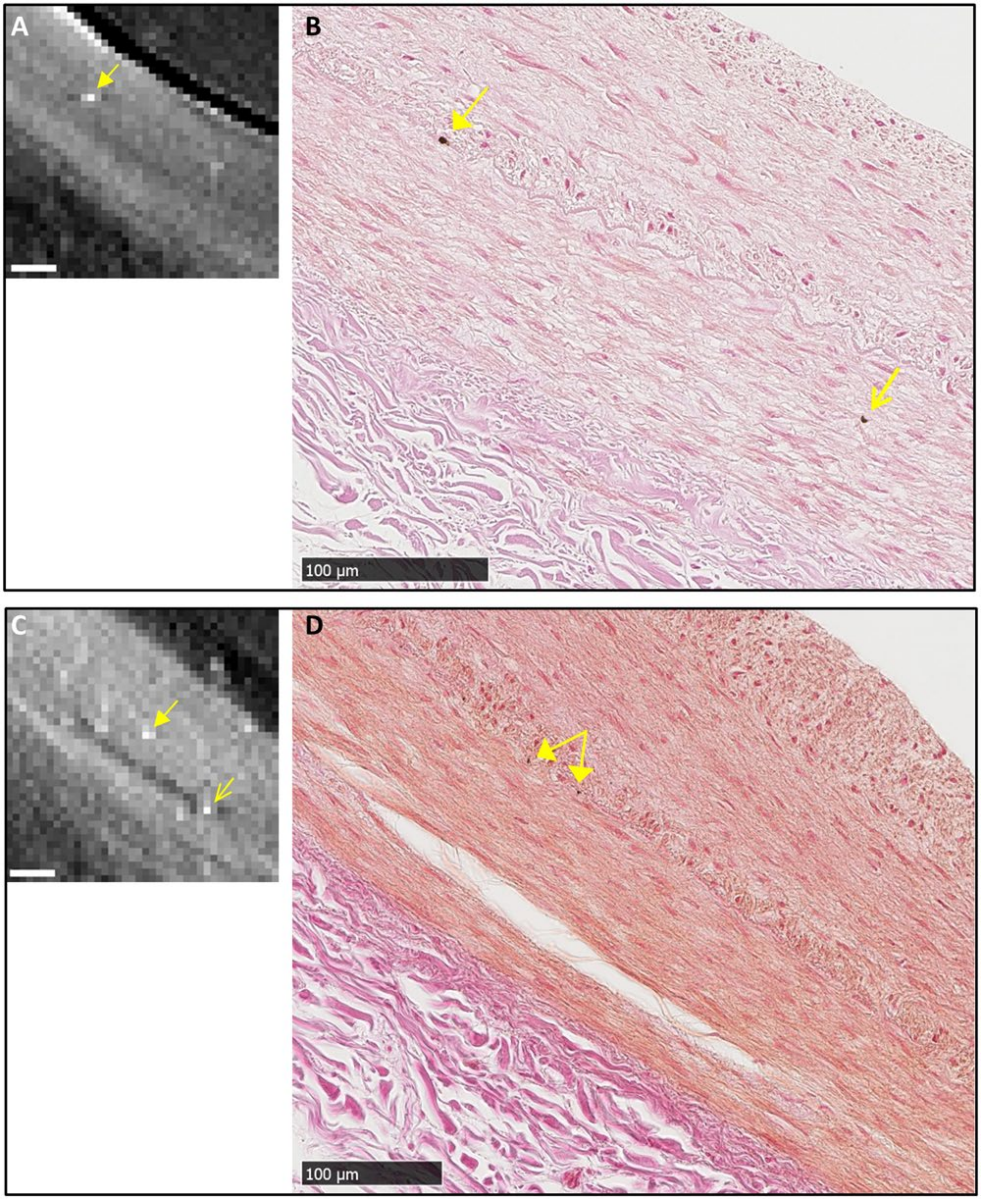
Two instances of un-correlated finds of micro calcifications in the media layer. (**A**) and (**B**) Near an IEL micro calcification visible in both tomography and histology (solid arrows), an isolated micro calcification in the media layer is seen in the histological image of panel B (open arrow), but not seen in the tomographic image of panel A. (**C**) and (**D**) Near focal calcifications in the IEL seen in both the tomographic and histological images (solid arrows), a focal calcification in the media is seen in the tomographic image (open arrow in panel C) but absent in the histological image panel D. Its location was determined based on a tear in the media visible in both images. All scalebars are 100 µm.

Overall, there were 3 matched locations of extended loose intimal calcifications and 11 matched locations of IEL calcifications that were seen in both types of images in the coronary segments. However, in an additional two instances, isolated focal calcifications were seen in the vicinity of other IEL calcifications by either the tomographic scan or histology, but the findings were not correlated (Fig. 5). In one instance (Fig. 5A), near an IEL calcification seen by both methods, a dark brown stain of 3.8 µm size was present in the tunica media in the histology image, but absent in the tomographic image. Because a residual stain was also seen in the same location in the adjacent histological slide, it was likely a tissue calcification instead of an introduced contaminant particle during slide production. In another instance (Fig. 5B), again near an IEL calcification seen by both methods, a bright dot (15 µm) was seen in the tomographic image, while no staining was seen in the histology image at that location. The location was determined to be in the tunica media at the end of a tear which was visible in both images. The reason for the mismatch relates to limitations of both methods, which are outlined in the Discussion section below.

## Discussion

In this study of human coronary artery samples, a histopathology-dedicated tomographic scanner provided scouting and complementary information to standard microscopy and enabled detection of isolated micro calcifications down to the cellular level. When compared with a commercial tabletop micro-CT scanner, it provided substantially better image quality in 1/11^th^ the scan time (15 minutes vs. 2.75 hours). The tomography scan turned the histology process from a blanket search to a targeted search. A particular significance of the Tomopath complementary information in this study is that it enabled the detection of isolated and focal micro-calcification in the IEL which could not be ascertained by histology alone.

In this patient, a prior clinical CT scan had detected coronary artery disease^36^. The patient also had HIV related kidney disease. Generally, both intimal and medial calcifications are known to result from chronic kidney disease^37–39^. While intimal micro calcification often occurs in occlusive atherosclerotic lesions and contributes to their instability and rupture^40^, calcification in the internal elastic lamina is thought to be universally associated with calcification in the medial layer (Mönckeberg’s sclerosis) and contributes to the stiffening of the artery in the absence of atherosclerotic occlusive lesions^41^. However, in this patient, isolated and focal micro calcifications were seen predominantly in the IEL while occasionally in the tunica media. The IEL calcifications did not expand into the tunica media. Therefore, we speculate that these focal calcifications are the initiation of medial calcification in this patient.

The examples of mismatched observation between tomography and histology showed limitations of both methods. With the tomographic scan, the detection threshold of calcification was estimated to be 38 mg/ml of hydroxyapatite, or 1.2% of the solid crystal density, based on material attenuation values at 23 keV and a detection threshold equivalent to 4 times the standard deviation of the signal in the vessel wall (P = 3.2E-5). Small or low-density calcifications that resulted in an average density in a pixel below the threshold would not be detected with this level of confidence. This was likely the reason for tomography to miss some calcifications seen in histology. On the histology side, it has been documented in the literature that micro calcifications are washed out of specimens during sectioning and staining, resulting in loss of detection by histology^42–45^; additionally, dark spots in the histology images can be particle contaminants introduced during slide processing if they are not corroborated by the tomography scan of the intact sample.

On the technological front, although tomographic scanners for intraoperative breast specimen imaging are commercially available (for example, KUB Technologies Inc), they have insufficient resolution for histopathology. A dedicated scouting scanner for histopathology, which can approach single-cell resolution in minutes, is shown to be feasible based on a design that optimizes the flux and magnification factor of the sample, coupled with an efficient x-ray camera. Besides the benefit of time and labor savings, our study showed that it provides useful complementary information in some cases to standard pathology analysis. The results support a continued effort to develop a compact device for routine use in pathology labs.

## Methods

### Preparation of the human coronary artery samples

The left anterior descending (LAD) coronary artery of a deceased HIV patient who had been suspected of HIV-related cardiovascular disease was dissected and fixed in 10% buffered formalin and was processed with Leica ASP-300 tissue processor. Eight segments of the artery were embedded in two paraffin blocks using standard medium-depth embedding cassettes. This process rendered the specimens innocuous and suitable for storage at room temperature over time.

### X-ray tomographic scans

The sample paraffin blocks were first scanned with a prototype imaging device designed specifically for histopathology samples (Tomopath), and then with a commercial tabletop micro-CT scanner (Bruker Skyscan 1172). Design features of the Tomopath scanner included close proximity of the sample to the x-ray focal spot to increase the photon flux through the sample and magnification, and a photon counting camera to improve detection efficiency. The scanner consists of a commercial micro-focus x-ray source (Oxford Nova600) operating at 50 kV/6 W with a focal spot size of 10 µm and a photon-counting x-ray camera (Dectris Pilatus 100 k) of 487 by 195 image matrix and 172 µm pixel size covering an image area of 83.7 mm wide and 33.5 mm tall. The samples were positioned between the source and the detector to provide a projection magnification of 10 to 14 over the 5 millimeter thickness of the paraffin block. The camera imaging speed was 30 frames/second. Given the limited image height of the camera, 7 horizontal bands at incremental vertical positions were scanned to cover the height of the paraffin block, with 10% overlap between adjacent bands. The total scan time was 15 minutes. The raw data were reconstructed into cross-sectional images of 15 µm pixel size and 30 µm slice thickness.

The scan parameters on the commercial micro-CT system were x-ray tube setting of 29 kV/6 W, camera matrix of 3000 by 2096, sample rotation step of 0.11°, 1852 projections over 203.72° rotation angle at 1.77 sec exposure per projection, and total scan time of 2 hours 45 minutes. Image reconstruction included an under-sampling factor of 5 to improve signal-to-noise ratio. Reconstructed image pixel size was 30.9 µm.

### Sectioning, staining and optical microscopy of the histological slides

From the cross-sectional images provided by the Tomopath scan, the locations of micro calcifications were identified and their levels below the surface of the paraffin block were recorded. The block was then sectioned with a microtome to those levels of interest. The 5 to 10 micrometer-sections of interest were cut and mounted in positively charged slides. These slides were stained with either hematoxylin and eosin (H&E) for soft tissue structure, or von Kossa stain to highlight calcium phosphate deposits while also delineate the structural layers of the wall. The slides were then scanned in a Hamamatsu digital slide scanner (NanoZoomer Res 2.0) to obtain color microscopy images. The von Kossa stain was verified in a positive control slide of a mouse embryo section containing bone structures.

Level matching between the Tomopath reconstruction slice thickness of 30 µm and slice levels given by the microtome was verified in a separate calibration procedure that matched the Tomopath images with histological slides of known levels using visible landmarks. The calibration procedure used a linear regression between the two methods to obtain a level spacing of 31.16 ± 0.85 µm for adjacent tomographic slices (R^2^ = 0.992), which was in agreement with the Tomopath reconstruction parameter of 30 µm.

### Data Availability

All data generated or analyzed during this study are included in this published article and its Supplementary Information files.

### Ethical approval and informed consent

All experimental protocols were approved by the local IRB at the National Institutes of Health; the methods were carried out in accordance with the relevant guidelines and regulations; informed consent was obtained from all participants.

## Electronic supplementary material

Supplementary Movie S1

Supplementary Movie S2

Supplementary Video Information
