# Supplementary material for: Correlative Detection of Isolated Single and Multi-Cellular Calcifications in the Internal Elastic Lamina of Human Coronary Artery Samples
Source: Sci Rep. 2018 Jul 20;8:10978. doi: 10.1038/s41598-018-29379-6 (PMC6054664; doi:10.1038/s41598-018-29379-6)
Supplement: Supplementary file 3 — Supplementary Video Information [file 41598_2018_29379_MOESM3_ESM.docx]

Correlative Detection of Isolated Single and Multi-Cellular Calcifications in the Internal Elastic Lamina of Human Coronary Artery Samples

Han Wen^a,*^, Alejandro Morales-Martinez^b^, Houxun Miao^a^, Thomas C. Larsen^a^, Catherine P. Nguyen^a^, Eric E. Bennett^a^, Kellan P. Moorse^a^, Zu-Xi Yu^a^, Alan T. Remaley^a^, Manfred Boehm^a^, Ahmed M. Gharib^c^

^a^ National Heart, Lung and Blood Institute, National Institutes of Health, Bethesda, MD 20892

^b^ Department of Bioengineering, University of California, Berkeley, CA 94720

^c^ National Institute of Diabetic and Digestive and kidney Diseases, National Institutes of Health, Bethesda, MD 20892

^*^To whom correspondence should be addressed. E-mail: wenh@nhlbi.nih.gov

**Supplementary Movie S1** Serial cross-sectional images from the surface of the paraffin block to a depth of 2.8 mm in 30 µm steps, acquired in a 15 minute scan with the Tomopath tomographic scanner, a prototype dedicated scouting tool for histopathology protocols. To reduce video file size the images were under-sampled by a factor of 2.

**Supplementary Movie S2** Serial cross-sectional images from the surface of the paraffin block to a depth of 2.8 mm in 30 µm steps, acquired from a commercial tabletop micro-CT scanner (Bruker Skyscan 1172) in a 2.75 hour scan. To reduce video file size the images were under-sampled by a factor of 2.
